# Prevalence and risk factors of hypertension in rural Bangladesh: A population-based cross-sectional study

**DOI:** 10.1371/journal.pone.0351569

**Published:** 2026-07-10

**Authors:** Sobiya Aziz Badat, C. Emdad Haque, Shakhawat Hossain, Alan Katz, Gias U. Ahsan

**Affiliations:** 1 Natural Resources Institute, University of Manitoba, Winnipeg, Manitoba, Canada; 2 Department of Mathematics and Statistics, University of Winnipeg, Winnipeg, Manitoba, Canada; 3 Department of Family Medicine, Max Rady College of Medicine, University of Manitoba, Winnipeg, Manitoba, Canada; 4 Department of Public Health, Daffodil International University, Dhaka, Bangladesh; PLOS: Public Library of Science, UNITED KINGDOM OF GREAT BRITAIN AND NORTHERN IRELAND

## Abstract

Non-communicable diseases (NCDs), such as hypertension, are amongst the most fatal conditions afflicting people living in low- and middle-income countries (LMIC), including Bangladesh. This study addresses the lack of population-based studies in rural Bangladesh by examining the prevalence and distribution of hypertension and its associated risk factors. To this end, we surveyed adults aged ≥18 years (i.e., household heads and their spouses) from 7384 households across 149 villages in rural Bangladesh in 2017 using a semi-structured questionnaire to collect data on blood pressure, anthropometric, socioeconomic, lifestyle, and behavioral risk factors. Multivariate logistic regression analyses identified age, gender, and socioeconomic status as potential predictors of hypertension. The findings also showed that men and women from higher socioeconomic status (SES) groups had higher rates of overweight and obesity, risk factors for the development of hypertension (4.29% and 1.4% in adult men; 5.8% and 2.29% in adult women), as well as higher rates of fruit and vegetable consumption (10.29% and 7.25% in adult men; 9.95% and 6.84% in adult women). A significant association between tobacco consumption and age was observed for women (p=<0.0001), while higher levels of physical activity were found among men aged 45–54 years [OR:1.9, CI 95% (1.1–3.1)]. Furthermore, women in the highest SES brackets were 1.3 times as likely to engage in “moderate” physical activity as those in the lowest brackets. Age and overweight/obesity were found to be the strongest risk factors for hypertension in both genders, while education was not found to be significantly associated with hypertension in women. Notably, the findings revealed that 33.7% of men and 28.6% of women had elevated blood pressure, qualifying them as either prehypertensive or hypertensive. As such, we recommend that policy interventions aimed at stemming the growth of hypertension among Bangladesh’s rural populations should take gender-specific risk factors, rural-urban disparities, and socioeconomic context into serious policy consideration.

## Introduction

Non-communicable diseases (NCDs) cause premature deaths in 41 million people each year [1], accounting for a staggering 74% of mortality worldwide [2]. The higher prevalence of NCDs in low- and middle-income countries (LMIC) [3,4], particularly their outcomes, pose a threat with respect to economic sustainability [5]. Most NCDs in these countries are associated with common preventable risk factors related to four main behavioral variables: tobacco use, physical inactivity, unhealthy diet, and excessive alcohol use [6–11]. These behavioral risk factors commonly result in five key metabolic/physiological changes: raised blood pressure (hypertension); being overweight/obese; raised blood glucose (diabetes); elevated cholesterol; and hyperlipidemia [12].

In the context of resource-constrained settings, the lack of hypertension detection [13] and discrepancies between physician-perceived and calculated risk (e.g., using a standardized scoring system for cardiovascular risk) are concerns for health policymakers [12]. Regarding such discrepancies, Cesaro et al. [12] observed that physician-perceived cardiovascular risk was underestimated in 37% of cases. They argued that this discrepancy underscores a potential gap in clinical practice, indicating that reliance on clinical judgement alone may be insufficient for accurate risk estimation and should be complemented by an evidence-based risk score.

In a recent systematic meta-analysis, Ranzani et al. [14] observed that hypertension in LMICs increased during 1990–2020 in both urban and rural areas, with a stronger trend in rural areas. In this context, the multifaceted disparities in rural vs. urban healthcare facilities, infrastructure, and socioeconomic conditions have been highlighted by numerous studies, including Huang et al. [15], Zeba et al. [16], and Zhang et al. [17]. Furthermore, the growing ‘urbanicity’ of the countryside, driven by the rapid adoption of lifestyle changes among the rural population, is contributing to the epidemiological shift towards NCDs (including hypertension) in the LIMICs [18,19], particularly in Bangladesh [20,21].

Bangladesh is an LMIC in South Asia, home to more than 170 million people. Research focusing on NCDs in Bangladesh is important, as the country is undergoing an “epidemiological transition” wherein chronic non-communicable degenerative conditions such as hypertension, diabetes, and obesity are rapidly displacing infectious diseases as the major cause of death [6,7,10,22–24]. Hypertension is one of the leading causes of death in Bangladesh [5], with many such deaths being premature [6,7]. To compound the problem, the prevalence of hypertension in Bangladesh was projected to grow from 26% in 2000 to 29% in 2025 [25]. Additionally, hypertension and diabetes share a complex interconnection, with both being high-risk factors for heart disease [6,8,9]. The literature on the role of hypertension in the development of coronary artery disease and myocardial ischemic syndromes, and other cardiovascular diseases is voluminous. Elaborating on this relationship, Volpe and Gallo [26] noted that the associations between hypertension and coronary artery disease are complex, involving overactivation of neurohormones, accelerated atherosclerotic plaque development, endothelial dysfunction, and altered intramyocardial coronary circulation. Furthermore, a nationwide cohort study in Taiwan found that both type 2 diabetes mellitus with subsequent hypertension and hypertension with subsequent diabetes were associated with higher cardiovascular disease risk [27].

Bangladesh has a higher comorbidity of diabetes-hypertension compared to other LMICs [6,8,24] and, by extension, a greater risk for heart disease. Recent studies have confirmed that the prevalence of hypertension in Bangladesh is on the rise among adults aged 35 years and older [9,28–30], while others have identified an increase in risk factors, such as lifestyle and behavioral factors (e.g., obesity, diet, and physical activity levels), among both men and women [6,8,28,31–34]. Indeed, findings have revealed a significant association between obesity/overweight and hypertension [6,31,35,36] and have identified physical activity as an important risk factor [6–8,10] for hypertension. In LMICs, differential stages of demographic and nutritional transitions have been found to be associated with hypertension prevalence [9,13,37–42]. Several studies in Bagladesh have also demonstrated that socioeconomic status and inequalities in wealth distribution pose significant risk factors for prehypertension and hypertension [6–8,10,24,43], and that these risk factors vary based on place of residence, educational attainment, occupational level, and tobacco use. The STEPwise Surveillance for NCD risk factors (STEPS) survey of 2018 and two nationally representative datasets, namely, the Bangladesh Demographic and Health Survey (BDHS) 2011 and 2017–18, have highlighted the growing prevalence of NCDs and various risk factors [9,44]. However, the literature on these subjects exhibits an urban bias, with very little research examining the rural population. Furthermore, existing studies typically do not consider the broader population and are instead targeted to a specific area, age group, or gender [32,35,43,45,46]. Moreover, studies examining and comparing the association between certain risk factors for hypertension are also rare. While precisely measuring hypertension and its risk factors in the general population is challenging, access to such data is critically important, as it would help researchers and policymakers understand rates and patterns of hypertension and, thus, how to formulate effective policy interventions.

Bangladesh has high rates of hypertension that are associated with risk factors such as dietary habits and lifestyle. Considering that hypertension is one of the most important NCDs in Bangladesh, this study investigates its distribution patterns among rural populations, with a particular focus on physical, behavioral, and dietary risk factors. This research aims to achieve two specific objectives: i) to identify and analyze the physical, behavioral, and dietary risk factors associated with prehypertension and hypertension among adult males and females in rural Bangladesh, and ii) to determine the magnitude of the effects of the identified risk factors upon prehypertension and hypertension among this cohort.

## Methods

### Data sources, study population, and survey design

A multi-stage sampling method was used to select 149 villages across eight districts of Bangladesh: Moulvibazar, Sunamganj, Sherpur, Jamalpur, Munshiganj, Pabna, Satkhira, and Khulna. Two Upazilas (sub-districts) were randomly selected from each district, for a total of 16 Upazilas, from which villages were sampled. As the study was observational and involved no intervention, a control group was not used. This sample comprised 12% of the rural population across Bangladesh’s 64 districts, with the household as the *Primary Sampling Unit* (PSU). The following formula was used to calculate the optimal sample size (n) for the study:


$$\begin{array}{c} n=\text{Design Effect }\times \frac{\overset{\text{2}}{\underset{\text{1}-\frac{\alpha }{\text{2}}}{Z}}\;p\;q}{\delta^{\text{2}}} \end{array}$$ (1)

where $Z_{\text{1}-\frac{\alpha }{\text{2}}}$ is the $\text{1}-\frac{\alpha }{\text{2}}$ quantile of the standard normal distribution, δ is the margin of error, and p represents the proportion of the target indicator, q=1−p.

To ensure a confidence level of 95%, it was assumed that  α=0.05, $Z_{\text{1}-\frac{\alpha }{\text{2}}}=\text{1}.\text{9}\text{6},$ and the margin of error was 5% (i.e., δ=0.05). Finally, the design effect was set as 1, as the simple random sampling method was applied to select households within the village.

Since the value of p was unknown, it was assumed that p = 0.5 would yield the maximum number of samples appropriate for measuring multiple indicators. Ultimately, a sample size of n=  384 samples were deemed necessary for each sampling unit (i.e., rural Upazila) to achieve a 95% confidence interval. The rural areas consisted of 384 PSUs x 16 Upazilas, yielding a total required sample size of 6144 PSUs. This sample was then extrapolated to 7,384 to obtain better coverage. Sample respondents were recruited for a four-month period, starting from 20 August 2017 till 21 December 2017. Data collection took place between August and December 2017 through semi-structured interviews with household heads (HHs) and their spouses in each PSU.

The survey process consisted of two components: i) data collection using a semi-structured questionnaire, with informed consent (written and verbal with a witness where necessary), and ii) a physical examination (conducted with participant consent). The questionnaires, formulated in *Bangla* (Bengali), translated into English, and validated for analysis, were administered via direct interviews in *Bangla*. Details of these instruments are provided in the studies by Shahidullah et al. [47] and Badat [48]. The standardized physical examinations measured height, weight, blood pressure, and blood sugar/glucose levels. The equipment used in the examinations included measuring tapes, digital scales, blood pressure monitors, stethoscopes, and glucometers. Field organizers and Information Technology personnel from Bangladesh’s Centre for Natural Resource Studies (CNRS) provided necessary technical support and troubleshooting throughout our fieldwork. The questionnaire consisted of multiple modules, with the preliminary questionnaire being modified to incorporate additional questions based on pre-testing in communities outside the study areas.

### Outcome variable

Hypertension served as the primary outcome variable. Blood pressure was measured using standard aneroid sphygmomanometers, which were placed on the participant’s right arm (which was supported) for at least 5 minutes while resting in a seated position. The measurement was taken at the participant’s home premises. Korotkoff phase V was used to determine diastolic blood pressure, with systolic and diastolic blood pressure measured in mmHg. Physicians performed device calibration and quality-control checks for blood pressure measurements [47]. Prehypertension was defined as systolic blood pressure between ≥120 mmHg and <140 mmHg, or diastolic blood pressure between ≥80 mmHg and <90 mmHg. Hypertension was defined as systolic blood pressure ≥140 mmHg or diastolic blood pressure ≥90 mmHg [6,8,10,24,49]. These diagnostic criteria are consistent with multiple previous studies, including Fottrell et al. [8] and Biswas et al. [10]. Additionally, several other studies on hypertension have provided similar cut-offs;; these include the definition used in the Demographic Health Survey [6,8,10] and the 2017 American College of Cardiology/American Heart Association (ACC/AHA) recommended cut-offs [24]. Participants were also asked whether they were currently taking any anti-hypertensive medication. However, since this part of the survey was conducted in the context of disease management, we relied solely on blood pressure readings to classify individuals as hypertensive.

### Independent variables

Principal component analysis (PCA) was conducted to identify the key household assets related to wealth from a list of 38 assets. These assets included: house and land ownership; fruits and vegetables produced and sold; number of rooms in one’s house; wall materials; water sources; sanitation infrastructure; electricity supply; and ownership of household assets, such as refrigerators, televisions, sewing machines, bicycles, or motorcycles. The PCA results were used to calculate a “wealth quintile” score for each household, and households were assigned to one of five socioeconomic quintiles based on their score: “most poor,” “least poor,” “lower-middle,” “higher-middle,” and “rich.”

The independent variables were selected based on the existing literature on risk factors for hypertension in LMICs and Bangladesh. The following independent variables were selected: place of residence (rural); wealth status (as defined above); age of participants (18–34, 35–44, 45–54, 55–64, ≥ 65); sex (male and female); level of education (no schooling vs. formal education such as elementary, secondary (SSC, JSC), higher secondary and above (HSC and other degrees)); occupation (farming/fishing, day laborer/other labor, other service holders (village doctor, service, small business), unemployed and other non-wage earners (student, retired, unemployed, housewife); and consumption of tobacco and related products.

Body mass index (BMI) was measured based on the WHO’s definition of obesity for the Asian population [6]: underweight (BMI < 18.5 kg/m^2^), normal weight (BMI 18.5–23.0 kg/m^2^), overweight (BMI 23.0–27.5 kg/m^2^), and obese (BMI ≥ 27.5 kg/m^2^). Physical activity was measured by asking respondents how much time (in minutes) they spent per day and per week engaging in moderate and/or vigorous activity. Additionally, this portion of the survey inquired about activities related to work, leisure, transportation, and time spent on sedentary pursuits. Physical activity was categorized into “vigorous,” “moderate,” and “sedentary” activities. Vigorous activity refers to any activity that results in a considerable increase in breathing or heart rate when maintained for at least 10 minutes. Moderate activity refers to any activity that causes a nominal increase in breathing or heart rate if continued for at least 10 minutes [47]. These data were then used to calculate the Metabolic Equivalent of Task (MET) -minutes, which was performed using the following STEPS protocol [11]: (a) 1 minute in a sedentary position (sitting quietly) equals 1 MET-minute; (b) 1 minute engaging in moderate and transportation-related activities equals 4 MET-minutes; and (c) 1 minute engaged in vigorous activity equals 8 MET-minutes. Individuals’ physical activity levels were then classified as either high (≥ 3000 MET-minutes), moderate (600–3000 MET-minutes), or low (≤ 600 MET-minutes) based on their total MET-minutes.

Adequate intake of fruits and vegetables was defined according to the WHO [50] guidelines. In general, these guidelines define one standard serving of vegetables as 80 grams; one serving raw leafy green vegetables as one cup; one serving of cooked or chopped vegetables as one-half cup; one serving of fruit (apple, banana, orange) as equaling one medium-size piece; one serving of chopped, cooked, or canned fruit as one-half cup; and one serving of fruit juice as one half-cup.

### Statistical analysis

To investigate the risk factors associated with hypertension, we conducted a series of statistical analyses using SAS 9.4 (SAS Institute Inc., Cary, NC). First, we described the sociodemographic characteristics of the study population and the respondents’ household characteristics (Tables 1 and 2). Next, we calculated the prevalence of hypertension and examined its relationship with different age groups, genders, and wealth quintiles (Tables 3 and 4). A chi-square test was then employed to assess any statistically significant relationships. Finally, we conducted a multinomial logistic regression analysis using a stepwise selection method to determine how these risk factors, both independently and collectively, influenced the prevalence of hypertension (Table 5). Our response variables were pre-hypertensive vs. non-hypertensive and hypertensive vs. non-hypertensive. The odds ratio was used to quantify the strength of association between two categorical variables, with p-values <0.05 considered statistically significant throughout the analysis.

**Table 1:** Background characteristics of the respondents (n = 14,343).

Variables	(n)	(%)
AGE		
18-34	4263	29.72
35-44	3880	27.05
45-54	3174	22.13
55-64	1951	13.6
≥65	1075	7.49
SEX		
Male	7123	49.66
Female	7220	50.34
MARITAL STATUS		
Married	13954	97.29
Unmarried	44	0.31
Divorced/separated	71	0.49
Widowed	274	1.91
EDUCATION		
No schooling and formal education	6135	42.77
Elementary/primary (up to Grade 5)	5356	37.34
Secondary (6–10 grade)	2409	16.8
Higher secondary and above (Grade 11 and above)	443	3.09
OCCUPATION		
Farming and fishing	2252	15.7
Day labors and labors	2525	17.6
Other services	2053	14.31
Unemployed and other non-wage earners	7513	52.38
SOCIO-ECONOMIC STATUS (SES) INDEX (based on wealth quintile)		
Most poor	2630	20.07
Least poor	2604	19.88
Lower-middle	2607	19.9
Higher middle	2623	20.02
Rich	2637	20.13

**Table 2:** Household characteristics of respondents by age group and gender.

Male Household Heads (n = 7123)							
	Age (years)						
	18-34		35-44	45-54	55-64	65+	Total
EDUCATION							
No schooling & formal education	n (%)	390 (28.59)	746 (41.12)	827 (47.86)	691 (52.55)	519 (57.54)	3173 (44.55)
Elementary/primary (up to Grade 5)	n (%)	641 (46.99)	686 (37.82)	546 (31.60)	382 (29.05)	231 (25.61)	2486 (34.90)
Secondary (Grade 6–10)	n (%)	260 (19.06)	300 (16.54)	282 (16.32)	193 (14.68)	118 (13.08)	1153 (16.19)
Higher secondary & above (Grade 11 and above)	n (%)	73 (5.35)	82 (4.52)	73 (4.22)	49 (3.73)	34 (3.77)	311 (4.37)
OCCUPATION							
Farming & fishing	n (%)	291 (21.33)	471 (25.96)	554 (32.06)	550 (41.83)	352 (39.02)	2218 (31.14)
Day labor & labor	n (%)	614 (45.01)	664 (36.60)	569 (32.93)	320 (24.33)	157 (17.41)	2324 (32.63)
Other services	n (%)	368 (26.98)	592 (32.64)	510 (29.51)	325 (24.71)	129 (14.30)	1924 (27.01)
Unemployed & other non-wage earners	n (%)	91 (6.67)	87 (4.80)	95 (5.50)	120 (9.13)	264 (29.27)	657 (9.22)
MARITAL STATUS							
Currently married	n (%)	1304 (95.60)	1804 (99.45)	1720 (99.54)	1300 (98.86)	863 (95.68)	6991 (98.15)
**Total**	n (%)	1364 (19.15)	1814 (25.47)	1728 (24.26)	1315 (18.46)	902 (12.66)	7123 (100)
WEALTH QUINTILES (SES Status)							
Most poor	n (%)	291 (24.41)	348 (21.13)	298 (18.56)	211 (17.09)	156 (18.77)	1304 (20.03)
Least poor	n (%)	248 (20.81)	334 (20.28)	298 (18.56)	255 (20.65)	158 (19.01)	1293 (19.86)
Lower-middle	n (%)	229 (19.21)	313 (19.00)	315 (19.61)	265 (21.46)	169 (20.34)	1291 (19.83)
Higher middle	n (%)	213 (17.87)	327 (19.85)	352 (21.92)	235 (19.03)	173 (20.82)	1300 (19.97)
Rich	n (%)	211 (17.70)	325 (19.73)	343 (21.36)	269 (21.78)	175 (21.06)	1323 (20.32)
**Total**	n (%)	1192 (18.31)	1647 (25.30)	1606 (24.67)	1235 (18.97)	831 (12.76)	6511 (100)
**Female Spouses (n = 7220)**							
	**Age (years)**						
	**18-34**		**35-44**	**45-54**	**55-64**	**65+**	**Total**
EDUCATION							
No schooling & formal education	n (%)	505 (17.42)	937 (45.35)	914 (63.21)	472 (74.21)	134 (77.46)	2962 (41.02)
Elementary/primary (up to Grade 5)	n (%)	1454 (50.16)	832 (40.27)	417 (28.84)	132 (20.75)	35 (20.23)	2870 (39.75)
Secondary (Grade 6–10)	n (%)	850 (29.32)	271 (13.12)	103 (7.12)	29 (4.56)	3 (1.73)	1256 (17.40)
Higher secondary & above (Grade 11 and above)	n (%)	90 (3.10)	26 (1.26)	12 (0.83)	3 (0.47)	1 (0.58)	132 (1.83)
OCCUPATION							
Farming & fishing	n (%)	4 (0.14)	12 (0.58)	12 (0.83)	5 (0.79)	1 (0.58)	34 (0.47)
Day labor & labors	n (%)	54 (1.86)	76 (3.68)	50 (3.46)	17 (2.67)	4 (2.31)	201 (2.78)
Other services	n (%)	42 (1.45)	41 (1.98)	33 (2.28)	11 (1.73)	2 (1.16)	129 (1.79)
Unemployed & other non-wage earners (including housewives)	n (%)	2799 (96.55)	1937 (93.76)	1351 (93.43)	603 (94.81)	166 (95.95)	6856 (94.96)
MARITAL STATUS							
Currently married	n (%)	2876 (99.21)	2008 (97.19)	1359 (93.98)	582 (91.51)	138 (79.77)	6963 (96.44)
**Total**	n (%)	2899 (40.15)	2066 (28.61)	1446 (20.03)	636 (8.81)	173 (2.40)	7220 (100)
WEALTH QUINTILES (SES STATUS)							
Most poor	n (%)	608 (23.41)	343 (17.95)	240 (17.76)	106 (18.40)	29 (18.71)	1326 (20.12)
Least poor	n (%)	520 (20.02)	379 (19.83)	274 (20.28)	102 (17.71)	36 (23.23)	1311 (19.89)
Lower middle	n (%)	502 (19.33)	391 (20.46)	267 (19.76)	120 (20.83)	36 (23.23)	1316 (19.97)
Higher middle	n (%)	505 (19.45)	403 (21.09)	277 (20.50)	114 (19.79)	24 (15.48)	1323 (20.08)
Rich	n (%)	462 (17.79)	395 (20.67)	293 (21.69)	134 (23.26)	30 (19.35)	1314 (19.94)
**Total**	n (%)	2597 (39.41)	1911 (29.00)	1351 (20.50)	576 (8.74)	155 (2.35)	6590 (100)

**Table 3:** Prevalence and ORs of risk factors of hypertension by age in rural Bangladesh.

		MALE					FEMALE				
		18-34	35-44	45-54	55-64	65+	18-34	35-44	45-54	55-64	65+
**Physical Activity**		p=<0.0001*					p=<0.0001*				
Moderate physical activity	%	1.97	3	3.54	2.88	3.16	4.32	4.31	3.3	1.94	0.79
	OR (95% CI)	1	1.7 (1.1-2.9)	2.7 (1.6-4.7)	1.7 (1.0-2.8)	1.3 (0.8-2.0)	1	1.2 (0.8-1.9)	1.3 (0.8-2.1)	0.8 (0.5-1.2)	0.7 (0.4-1.4)
High physical activity	%	16.65	22	20.37	15.12	8.83	5.04	3.67	6.27	6.4	1.41
	OR (95% CI)	1	1.5 (0.9-2.4)	1.9 (1.1-3.1)	1.0 (0.7-1.7)	0.4 (0.3-0.6)	1	0.8 (0.6-1.2)	0.8 (0.5-1.2)	0.3 (0.2-0.5)	0.2 (0.1-0.3)
**Body Mass Index**		p=<0.0001*					p=<0.0001*				
Underweight	%	5.49	6.51	6.36	5.38	4.27	9.39	5.43	4.27	2.44	0.61
	OR (95% CI)	1	0.95 (0.8-1.1)	0.9 (0.8-1.1)	1.1 (0.9-1.3)	1.3 (1.1-1.6)	1	0.9 (0.8-1.0)	0.9 (0.8-1.1)	1.2 (0.9-1.5)	1.0 (0.7-1.5)
Overweight	%	3.06	5.18	4.96	3.45	2.16	9.85	8.75	5.22	1.95	0.61
	OR (95% CI)	1	1.4 (1.1-1.6)	1.4 (1.1-1.7)	1.3 (1.0-1.6)	1.2 (0.9-1.6)	1	1.4 (1.2-1.6)	1.1 (0.9-1.3)	0.9 (0.7-1.1)	1.0 (0.7-1.5)
Obese	%	0.7	1.43	1.12	0.86	0.52	4.03	3.5	2.63	0.76	0.14
	OR (95% CI)	1	1.6 (1.2-2.3)	1.3 (0.9-1.9)	1.4 (0.9-2.0)	1.3 (0.8-1.9)	1	1.3 (1.1-1.6)	1.4-1.1-1.7)	0.8 (0.6-1.1)	0.6 (0.3-1.1)
		p=<0.0001*					p=<0.0001*				
**Inadequate (“Too low”) vegetable intake**	%	6.21	9.08	9.48	7.78	4.84	13.4	10.82	8.57	3.19	0.8
	OR (95% CI)	1	1.2 (0.9-1.3)	1.4 (1.2-1.6)	1.5 (1.3-1.8)	1.3 (1.1-1.6)	1	1.2 (1.1-1.4)	1.5 (1.3-1.7)	1.1 (0.9-1.4)	1.0 (0.7-1.4)
		p=<0.0001*					p=<0.0001*				
**Inadequate (“Too low”) fruit intake**	%	3.54	4.9	5.66	4.83	3.24	7.51	6	5.35	2.2	0.71
	OR (95% CI)	1	1.1 (0.9-1.3)	1.4 (1.1-1.6)	1.6 (1.3-1.9)	1.5 (1.2-1.9)	1	1.2 (1.0-1.3)	1.6 (1.3-1.8)	1.4 (1.2-1.8)	1.8 (1.3-2.6)
		p = 0.0117					p=<0.0001*				
**Tobacco consumption**	%	11.23	15.39	15.39	11.85	7.99	15.1	13.8	10.79	5.24	1.44
	OR (95% CI)	1	1.1 (0.9-1.2)	1.2 (1.1-1.4)	1.3 (1.2-1.5)	1.2 (1.0-1.4)	1	1.5 (1.4-1.7)	1.9 (1.7-2.1)	2.4 (2.0-2.9)	2.5 (1.8-3.4)

**Table 4:** Prevalence and ORs of risk factors of hypertension by wealth quintile in rural Bangladesh.

		MALE					FEMALE				
		Most poor	Least poor	Lower middle	Higher middle	Rich	Most poor	Least poor	Lower middle	Higher middle	Rich
**Physical Activity**		p = 0.0241					p = 0.0218				
Moderate physical activity	%	2.58	2.52	3.21	3.07	3.12	2.53	2.59	3.13	3.17	3.13
	OR (95% CI)	1	0.6 (0.3-1.0)	1.0 (0.6-1.8)	1.1 (0.6-2.0)	1.1 (0.6-2.0)	1	0.7 (0.4-1.1)	1.0 (0.6-1.7)	1.3 (0.8-2.4)	1.3 (0.7-2.3)
High physical activity	%	7.05	16.66	16.14	16.48	16.77	17.15	16.62	16.3	16.49	16.4
	OR (95% CI)	1	0.6 (0.4-0.9)	0.8 (0.5-1.3)	0.9 (0.5-1.6)	0.9 (0.5-1.6)	1	0.6 (0.4-1.0)	0.8 (0.5-1.3)	1.0 (0.7-1.8)	1.0 (0.6-1.7)
**Body Mass Index**		p=<0.0001*					p = 0.1080				
Underweight	%	6.16	5.94	5.33	5.42	4.53	4.32	4.72	4.23	4.39	3.64
	OR (95% CI)	1	1.0 (0.9-1.2)	0.9 (0.7-1.0)	0.9 (0.7-1.0)	0.7 (0.6-0.8)	1	1.1 (0.9-1.4)	1.0 (0.8-1.2)	1.0 (0.8-1.2)	0.8 (0.7-1.0)
Overweight	%	3.33	3.87	4.33	3.82	4.29	5.52	5.02	5.2	5.4	5.8
	OR (95% CI)	1	1.2 (1.0-1.5)	1.3 (1.0-1.6)	1.1 (0.9-1.4)	1.2 (1.0-1.5)	1	0.9 (0.8-1.1)	0.9 (0.8-1.1)	0.9 (0.8-1.1)	1.0 (0.8-1.2)
Obese	%	0.91	0.92	0.65	1	1.4	2.28	2.43	2.5	2.06	2.29
	OR (95% CI)	1	1.0 (0.7-1.6)	0.7 (0.5-1.1)	1.1 (0.8-1.6)	1.5 (1.0-2.0)	1	1.1 (0.9-1.4)	1.1 (0.8-1.4)	0.9 (0.7-1.1)	0.9 (0.8-1.3)
		p=<0.0001*					p=<0.0001*				
**Inadequate (“Too low”) vegetable intake**	%	5.85	7.05	7.63	8.14	10.29	5.87	7.03	7.6	7.95	9.95
	OR (95% CI)	1	1.4 (1.2-1.6)	1.6 (1.3-1.8)	1.7 (1.5-2.0)	2.6 (2.2-3.0)	1	1.3 (1.1-1.6)	1.5 (1.3-1.8)	1.6 (1.4-1.9)	2.5 (2.1-2.9)
		p=<0.0001*					p=<0.0001*				
**Inadequate (“Too low”) fruit intake**	%	2.98	4.04	4.19	4.81	7.25	3	4.07	4.22	4.69	6.84
	OR (95% CI)	1	1.5 (1.2-1.8)	1.5 (1.2-1.9)	1.8 (1.5-2.2)	3.2 (2.7-3.9)	1	1.5 (1.2-1.8)	1.5 (1.2-1.9)	1.7 (1.4-2.1)	3.0 (2.5-3.6)
		p = 0.0213					p = 0.1419				
**Tobacco consumption**	%	12.19	11.96	12.27	12.82	11.78	8.92	9.09	9.09	9.82	8.92
	OR (95% CI)	1	0.9 (0.8-1.1)	1.0 (0.9-1.2)	1.2 (1.0-1.4)	0.9- (0.8-1.0)	1	1.1 (0.9-1.2)	1.0 (0.9-1.2)	1.2 (1.1-1.4)	1.0 (0.9-1.2)

**Table 5:** Logistic regression model identifying risk factors influencing hypertension in rural Bangladesh.

	Male			Female		
	Pre-hypertensive vs non-hypertensive		Hypertensive vs non-hypertensive	Pre-hypertensive vs non-hypertensive		Hypertensive vs non-hypertensive
		OR (95% CI)	OR (95% CI)		OR (95% CI)	OR (95% CI)
**AGE**	P=<0.0001*			P=<0.0001*		
18-34		1	1		1	
35-44		0.9 (0.8 - 1.1)	1.8 (1.1- 2.9)		1.3 (1.1- 1.5)	2.2 (1.6 - 3.0)
45-54		1.1 (0.9 - 1.3)	3.0 (1.9 - 4.8)		1.9 (1.6 - 2.2)	5.3 (3.9 - 7.2)
55-64		1.3 (1.1- 1.6)	5.4 (3.4 - 8.7)		2.9 (2.3 - 3.6)	10.4 (7.4 −14.6)
65+		1.7 (1.4 - 2.2)	9.5 (5.9 - 15.1)		2.7 (1.8 - 4.2)	12.9 (7.9 - 21.0)
**BODY MASS INDEX**	P=<0.0001*			P=<0.0001*		
Underweight		0.6 (0.5 - 0.7)	0.6 (0.4 - 0.8)		0.6 (0.5 - 0.7)	0.9 (0.7 - 1.2)
Normal		1	1		1	1
Overweight		2.1 (1.8 - 2.9)	2.9 (2.3 - 3.8)		1.9 (1.6 - 2.2)	1.9 (1.5 - 2.5)
Obese		2.2 (1.7 - 2.9)	3.8 (2.6 - 5.7)		2.8 (2.3 - 3.4)	4.2 (3.1 - 5.6)
**INADEQUATE (“TOO LOW”) FRUIT INTAKE**	P=<0.0001*			P=<0.0001*		
Yes		1.7 (1.5 −1.9)	1.6 (1.3 - 2.1)		1.5 (1.3 - 1.7)	1.4 (1.1 - 1.7)
No		1	1		1	1
**TOBACCO CONSUMPTION**	P = 0.0003			P = 0.0229		
Yes		0.8 (0.7 - 0.9)	0.8 (0.7 - 1.0)		0.9 (0.8 - 1.0)	0.8 (0.6 - 0.9)
No		1	1		1	1
**OCCUPATION**	P=<0.0001*					
Farming & fishing		0.9 (0.7 - 1.1)	0.5 (0.3 - 0.6)			
Day labor & labor		1.0 (0.8 - 1.2)	0.4 (0.3 - 0.6)			
Other services		1.1 (0.9 - 1.4)	0.6 (0.5 - 0.9)			
Unemployed & other non-wage earners		1	1			
**EDUCATION**	P = 0.0015					
No schooling & formal education		1	1			
Elementary/primary (up to Grade 5)		0.9 (0.9 - 1.1)	1.3 (1.0 - 1.7)			
Secondary (Grade 6–10)		1.1 (0.9 - 1.3)	1.8 (1.4–2.5)			
Higher secondary & above (Grade 11 and above)		1.4 (1.0–1.8)	1.7 (1.2–2.8)			

*Significant at P < 0.05 level.

### Ethics statement

The research and data collection protocols were approved by the Human Research Ethics Board at the University of Manitoba, Canada [Protocol Numbers: J2026:113 (HS20132) and HE2023–0279)] as well as by Bangladesh Medical Research Council’s National Research Ethics Committee (Protocol/Registration number: 105-07-03-2018). Data for the study were collected only after obtaining explicit written and verbal consent (in the presence of a witness) from all participants, in accordance with the ethics protocols of the University of Manitoba, Canada, and the Bangladesh Medical Research Council. Additionally, the procedures used in this study adhered to the principles outlined in the Declaration of Helsinki. Prior to data collection and obtaining informed consent, participants were provided with information about the study’s scope and objectives and notified that they had the right to withdraw from the study at any time, for any reason. A debriefing session was also held with the participants to share the preliminary results of this research.

## Results

### Sociodemographic characteristics

In total, 14745 respondents from 7384 households were surveyed, and 14343 participants (97.3% response rate) provided complete responses. Of these respondents, 49.6% (n = 7123) were male, and 50.3% (n = 7220) were female. Table 1 presents the sociodemographic characteristics of the study population. The distribution of the sample indicated a potential age bias, with over 56% of respondents aged 18–44 years and only 7.5% aged 65 years or older. Furthermore, more than 80% of the study population had received minimal or no formal education, with 42.7% having no formal education and 37.3% having completed only elementary or primary education (i.e., up to class 5). Only 3% of the population had attained higher secondary qualifications.

The study population’s occupations were distributed almost evenly across economic sectors: farming and fishing, 15.7% (n = 2252); day laboring, 17.6% (n = 2525); and other services and small business ownership, 14.3% (n = 2053). Most respondents, 52.3% (n = 7513), were non-wage earners or housewives. In terms of marital status, 97.2% of the respondents were married; hence, 52.3% (n = 7,513) of the study population consisted of housewives, who were primarily non-wage earners. Regarding SES, 39.9% of the population fell into the “poor” category, and 39.9% into the middle category. Within this “middle” category, 19.9% qualified as being “lower-middle” SES. The remaining 20.1% of the population was classified as “rich.”

The sociodemographic characteristics of the respondents by sex (male n = 7123; female n = 7220), along with their corresponding household characteristics (n = 7384), are presented in Table 2. Regarding age, females in the sample tended to be younger on average, with 40.1% in the 18–34 age group (compared with 19.2% of males); in contrast, 49.7% of males were in the 35–44 or 45–54 age groups. There were no notable differences between sexes with respect to wealth quintiles, as the male and female respondents belonged to the same households. However, significant differences were observed in occupation. Whereas 9.2% (n = 657) of male household heads were unemployed, with 29.2% (n = 264) being 65 years or above, 94.9% (n = 6,856) of females were non-wage-earning housewives. The overwhelming majority of these housewives (96.5%; n = 2799) were aged 18–34. In terms of education, 44.5% (n = 3173) of male respondents and 41.0% (n = 2962) of female respondents had no formal schooling, indicating low overall levels of literacy and education. Indeed, the majority of male (51.1%) and female (57.2%) respondents had completed no more than Grade 10.

### Risk factors

We examined multiple potential risk factors for hypertension, including respondent characteristics, household attributes, and behavioral factors. Table 3 summarizes these self-reported risk factors, categorized by sex, wealth quintile, and age. Notably, male respondents reported higher levels of physical activity and lower prevalence of obesity and overweight. In contrast, female respondents consumed more fruits and vegetables but had significantly higher rates of overweight, particularly among younger women (e.g., those aged 34).

The results revealed that the prevalence of elevated blood pressure among men was 33.7%, compared with 28.6% among women (p < 0.0001), indicating a significant gender difference. These participants qualified as either prehypertensive or hypertensive. While men reported higher physical activity and lower rates of overweight and obesity, women reported higher fruit and vegetable intake.

Overweight prevalence in women was highest in the younger age group (34 years and below). Although overweight rates generally increased with SES for both sexes, the odds ratios for the “middle” wealth quintiles were statistically similar to those of the “poorest” quintile. Among women, the prevalence of underweight, overweight, and obesity decreased with age, demonstrating a complex relationship with BMI. In contrast, men showed a more consistent positive association between age and overweight; the odds of being overweight or obese were 1.3 to 1.4 times higher in older age groups compared to younger ones.

Men aged 45–54 years reported the highest physical activity levels (with odds 1.9 times higher than the low levels of physical activity), whereas activity levels declined with both age and SES in women. The youngest women (18–34 years) reported the highest activity levels, and wealthier women were 1.3 times more likely to report moderate physical activity than the poorest women.

Inadequate fruit and vegetable intake was strongly associated with both age and wealth quintiles. Higher SES was associated with substantially higher odds of inadequate consumption of both vegetables (2.6-fold higher in men and 2.5-fold higher in women for the richest group compared to the poorest) and fruits (3.2-fold higher in men and 3.0-fold higher in women). Similarly, respondents aged 45–54 were more likely to report inadequate vegetable intake (1.4-fold higher odds in men and 1.5-fold higher in women) than those in the youngest age group. Among women, higher odds of inadequate fruit intake were observed across all older age categories (45 and above), with the likelihood of inadequate consumption 1.6–1.8 times higher than in the youngest group. Tobacco use was more prevalent in older women (up to 2.5 times higher odds in the 65 + group compared to the 18–34 group); regarding SES, a significant association with tobacco consumption was observed in men (p = 0.0213), but no significant association was observed in women (p = 0.1419).

Bivariate analyses (Tables 3 and 4) identified associations with key risk factors. Physical activity, BMI, and inadequate fruit and vegetable intake were significantly associated with age (p < 0.0001 for all). Tobacco use was a significant risk factor by age for both sexes (p = 0.0117 for men, p < 0.0001 for women). When stratified by SES (Table 4), inadequate fruit and vegetable intake remained significantly distributed across groups for both sexes (p < 0.0001). While BMI was significantly associated with SES in men (p < 0.0001), no such significant association was found in women (p = 0.1080). Finally, physical activity was significantly associated with SES in both men (p = 0.0241) and women (p = 0.0218), while a significant association between tobacco use and SES was observed only in men (p = 0.0213) and not in women.

A stepwise multinomial logistic regression analysis, using AIC and BIC as model selection criteria, was employed to develop the final logistic regression model. This iterative process involved adding and removing predictor variables, including age, physical activity, BMI, vegetable intake, fruit intake, tobacco consumption, education, and occupation. For male respondents, stepwise regression identified age, BMI, fruit intake, tobacco consumption, occupation, and education as significant predictors of hypertension. For female respondents, the analysis identified age, BMI, fruit intake, and tobacco consumption as significant predictors (see Table 4 in supplementary materials). In the odds ratio columns, “1” represents the reference category. Age was a particularly strong risk factor. After adjusting for all other variables in the model, men aged 65 + were 9.5 times as likely to have hypertension (CI: 5.9–15.1) compared with men aged 18–34, and those aged 45–54 were 3.0 times as likely (CI: 1.9–4.8). BMI also significantly impacted hypertension risk. Compared to men with normal weight, overweight men were 2.9 times as likely to have hypertension (CI: 2.3–3.8), while obese men were 3.8 times as likely (CI: 2.6–5.7), after adjusting for other variables.

After adjusting for all other factors, men with higher levels of education (higher secondary and above) were 1.7 times more likely to have hypertension than those with no formal education (CI: 1.2–2.8). Table 5 further examines the relationship between education level and hypertension. Men employed in the service sector had a lower risk of hypertension (OR 0.6; CI: 0.5–0.9) than unemployed and other non-wage earners.

Four factors significantly influenced hypertension among female respondents: age (p < 0.0001), BMI (p < 0.0001), fruit intake (p < 0.0001), and tobacco consumption (p = 0.0229). Age was a strong predictor. Compared to women aged 18−34, those aged 45−54 were 5.3 times more likely to have hypertension (CI: 3.9–7.2), and those aged 65 + were 12.9 times more likely (CI: 7.9–21.0), after adjusting for other factors. Obesity also significantly increased the risk. Obese women were 4.2 times more likely to have hypertension than women with normal weight (CI: 3.1–5.6), and overweight women were 1.9 times more likely (CI: 1.5–2.5), after adjusting for other factors. Finally, tobacco consumption was significantly associated with lower odds of hypertension; women who consumed tobacco were 0.8 times as likely to have hypertension as those who did not (CI: 0.6–0.9). This suggests a potential protective effect that warrants further investigation, which may be due to confounding variables.

## Discussion

The prevalence of hypertension among adults in low- and middle-income countries (LMICs) is substantial and has been steadily increasing over the past few decades. A systematic review and meta-analysis by Ranzani et al. [14] examined 299 population-based surveys from 66 LMICs, covering nearly 20 million adults aged 15 and older. The authors noted a pooled prevalence of hypertension of 30.5% in urban areas and 27.9% in rural areas between 1990 and 2020. The mean age of participants was approximately 45 years. Several global analyses have reported that the number of adults with hypertension nearly doubled from 1990 to 2019, with the majority of this increase occurring in LMICs due to population growth and aging. Despite this rise, awareness, treatment, and control rates remain low in many of these countries [13,51].

Our field investigation examined the prevalence and associated risk factors of hypertension (including physical, behavioral, dietary habits, and socioeconomic status) among individuals living in rural Bangladesh. Our findings indicated that one-third of respondents aged 18 years or older (33.7% of males and 28.6% of females) had elevated blood pressure, classified as either prehypertensive or hypertensive. This finding indicates a significant increase in the prevalence of hypertension among residents of rural Bangladesh, as the 2018 national STEPS survey only found 21% of respondents to be hypertensive [52]. Likewise, we found that the prevalence of hypertension had increased considerably compared to the WHO’s 2016 study [7], which found that diabetes and hypertension were prevalent among the rural adult population at a rate of 8% and 26.4%, respectively [7]. In recent decades, the findings of population-based studies have demonstrated a significant global increase in the prevalence of NCDs, particularly hypertension and diabetes. Similarly, previous studies have reported that hypertension has become a major health concern in Bangladesh [53], which they have ascribed to changes in dietary patterns, food insecurity, poverty, lifestyle factors, and urbanization [29,54–56].

Age has been consistently shown to be a significant risk factor for hypertension, with findings showing a direct correlation between age and hypertension prevalence [57–59]. For instance, one recent study found the prevalence of hypertension to be especially high among participants in the 50–53 age group [9]. Furthermore, findings suggest that, after the age of 59, hypertension rates tend to be higher among women compared to men [57–59]. Our results confirm these findings, revealing a steady increase in hypertension prevalence with age, particularly among older individuals in rural Bangladesh. Indeed, we observed the highest rates of hypertension in women aged 45–54 years and above 65 years. While previous studies have found lower hypertension rates among adults younger than 39 [10], our findings indicate a shift in this trend. In addition, we also find that individuals under 45 years old are both at greater risk of hypertension, with men and women being 1.8 and 2.2 times more likely to develop hypertension, respectively, compared to older age groups. Several recent studies attributed this pattern to changes in lifestyle, occupation and effects of urbanicity in the rural areas by the younger age group. [29,60,61]. Our findings align with Fottrell’s [6] data, which shows a high prevalence of hypertension in the elderly population (aged 60 and over). These results underscore the growing problem of hypertension among older age groups and emphasize the need for targeted interventions and preventative measures.

Gender disparities in hypertension rates have been well-established [5,57–59,62–65]. Our data indicated that the overall prevalence of hypertension differed significantly between sexes (33.7% of males and 28.6% of females were prehypertensive or hypertensive; p-value < 0.0001). This difference is due to the predominance of pre-hypertensive. It was further revealed that some of the risk factors related to hypertension among the sexes are also significant. This result is consistent with previous studies of Bangladesh, which have found a close association between risk factors such as age, BMI, occupation, tobacco consumption, and fruit and vegetable consumption and high blood pressure across genders [6,8,32,44]

Evidence from the 2017–18 Bangladesh Demographic and Health Survey (BDHS) indicates that the prevalence of hypertension increases markedly with age: approximately 39.4% of adults aged 35 and older are affected, compared with 12.2% among those aged 18–34 [66]. This trend aligns with global patterns and underscores the need for early age-targeted screening initiatives, ideally initiated before age 35. Such proactive approaches can help identify pre-hypertensive individuals, enabling timely lifestyle interventions and reducing the risk of long-term cardiovascular complications. Alarmingly, recent studies also report growing hypertension prevalence among younger adults, particularly those in urban and peri-urban regions. This shift is attributed to increasing sedentary behavior, high dietary sodium intake, and reduced physical activity, which are linked to occupational transitions and urbanization [60,61]. These findings suggest the need for youth-focused interventions, such as awareness campaigns, workplace wellness programs, and the integration of blood pressure checks into routine care services. Unlike trends in many high-income countries, where men typically exhibit a higher prevalence of the condition, BDHS data reveal that Bangladeshi women bear a higher burden of hypertension (29.5%) than men (26.2%)—especially women with low physical activity and limited access to health information [66]. This gap warrants gender-sensitive strategies, including mobile health clinics, female community health workers, and the integration of hypertension screening into reproductive and maternal health services. Conversely, studies show that although men may have a slightly lower prevalence, they are often underdiagnosed and less likely to adhere to treatment, primarily due to poor health-seeking behavior and limited engagement with primary health services [5].

Studies of LMICs have identified education as one of the most important risk factors for hypertension, with many demonstrating a strong inverse relationship between educational attainment and hypertension in women [29,30,45,59,67–70]. For instance, Rahman [7] found significantly higher rates of hypertension among women with little or no education. Interestingly, although 80.7% of the females in our sample had a maximum education level of Grade 5, our findings did not indicate a significant association between their level of education and prehypertension or hypertension rates. This finding contradicts the trend observed in other LMIC studies, including findings from studies in Bangladesh [28,29,31,36,45,52,71–73]. The patterns in our study are likely attributable to other confounding factors, such as age, socio-economic status, and BMI, similar to prior findings reported by Chowdhury et al. [28] and Zahangir et al. [74].

The impact of limited education on dietary choices cannot be ignored and remains a crucial factor in dietary and health outcomes. Previous studies of rural Bangladesh have found that female household heads tend to make healthier food choices for their families, and that their understanding of “healthy” and “unhealthy” diets is significantly influenced by their own educational background [33,74,75]. This lack of awareness, coupled with potential disparities in access to adequate quantities and quality of food, can negatively affect the health outcomes of women and their dependents [75–77], regardless of their control over household decisions.

In our study of rural Bangladesh, we found that across all age groups and socioeconomic levels, women were significantly more likely than men to consume adequate amounts of fruits and vegetables. Specifically, wealthy women were 2.5 times as likely to eat sufficient vegetables and 3 times as likely to consume sufficient fruit. In contrast, wealthy men exhibited a minimal increase, with odds ratios for adequate vegetable and fruit consumption rising by 1.7 and 1.8, respectively. In many cultures, food consumption patterns are highly symbolic, reflecting underlying power differentials along gender lines. For instance, diets that are high in meat are often perceived as masculine, while the consumption of fruits and vegetables is typically seen as being more feminine [77], which can be protective against hypertension. This gendered lens influences food availability and distribution, particularly in LMICs, where gender preference and bias can impact dietary intake from early childhood [78]. These factors, along with lifestyle and SES differences between urban and rural populations, can intensify the risk factors for NCDs in women [45].

For the male participants, the odds of being prehypertensive or hypertensive increased with higher educational attainment and employment in non-physical occupations. This aligns with previous studies of Bangladesh conducted by Sathi [9], Fottrell [6], and Kibria [24], whose findings indicate that educated populations experience higher rates of diabetes, hypertension, and obesity due to sedentary lifestyles. We also found a notable positive association between wealth and risk of obesity/overweight, as men who qualified as being “rich” were 1.2 and 1.5 times more likely to be obese and overweight, respectively, compared to the poorest respondents. Our findings align with those of Ali et al. [32], who also observed increased obesity and hypertension rates among Bangladeshi men from middle to higher SES backgrounds.

Similarly, being overweight or obese also significantly increased the odds of hypertension among women, with obese women being 4.2 times more likely to be hypertensive compared to those with normal body weight. In one study of young adults across various regions of Bangladesh, Ali [32] identified abdominal obesity as a major risk factor for hypertension in women, particularly among those living in Dhaka, Barisal, and Rangpur Divisions. However, as Zahangir [74] notes, underweight women are also at risk of hypertension. Research focusing on rural Bangladesh has revealed a unique trend wherein wives of migrant laborers, both in urban Bangladesh and abroad, tend to be overweight or obese rather than undernourished. This phenomenon has been attributed to these women’s increased purchasing power, which enables them to access higher-quality food and hire domestic help [79]. In addition, the literature indicates that women from higher-income households generally have lower levels of physical activity [9,31]. This trend is reflected in our results, which show a significant inverse relationship between SES and physical activity (p = 0.02) among the female participants. It is notable that reduced physical activity could be both cause and consequence of hypertension [10,32]. However, the association between SES and BMI (p = 0.10) was not found statistically significant in our study. This finding runs counter to those of previous studies showing a strong positive relationship between SES and overweight/obesity among female participants [6,24,31,32,74].

Tobacco use, including smoking and chewing, is a significant health burden in South Asian countries such as India, Pakistan, and Bangladesh, as such behavior contributes to oral and lung cancers, as well as increased rates of NCDs such as hypertension and cardiovascular diseases, particularly among men [70,79,80–83]. However, our results could not establish a positive association between tobacco consumption and hypertension among the rural adults in Bangladesh, which is likely due to Type II error (i.e., false-negative rejection of the null hypothesis). However, our findings showed a significant positive correlation between tobacco consumption and age among women, with older women (65+) being 2.5 times more likely to use tobacco products compared to women in the 18–34 age group.

Notably, a study by Rahman [46] found a strong association between cigarette smoking and hypertension in Bangladeshi men. While tobacco use has declined among younger generations in recent years, older men and women continue to engage in these habits. The social stigma associated with women using tobacco is likely a major reason for the higher rates of tobacco use observed among men compared to women [28,65]. Although our study revealed a significant association between SES and tobacco consumption (p = 0.02) in men, usage rates were found to be lower among men qualifying as “rich.” Similar results have been reported in previous studies, where findings show tobacco consumption being prevalent in all SES categories, but lower among higher SES cohorts [6,34,42].

Our study found no direct association between SES and hypertension prevalence. This diverges from the findings of numerous prior studies, which have consistently demonstrated a positive association between household wealth and hypertension [6–8,24,32,43]. However, our findings indicate that SES affects lifestyle choices, particularly physical activity and diet. Consistent with prior findings, we found that individuals from higher SES groups exhibited healthier dietary habits, including greater consumption of fruits and vegetables [6,74]. This suggests that, while lifestyle factors play an important role in risk for hypertension, other complex factors, such as age and gender, are likely to contribute to its prevalence [24]. Kirschbaum’s [5] study of 76 LMICs found that significant regional differences in NCD prevalence were largely driven by socioeconomic disparities. While increased per capita GDP generally correlated with decreased hypertension risk factors, particularly among men, this pattern did not hold true for South-Asian countries, where risk factors continued to rise alongside SES [24]. Our findings indicate significant socioeconomic inequality in the prevalence of hypertension risk factors, specifically physical activity levels, BMI, fruit and vegetable intake, and tobacco consumption, across genders and age categories among residents of rural Bangladesh.

For long-term disease control, these patterns reinforce the importance of monitoring systems that track hypertension prevalence and outcomes by age and sex. Community-based monitoring, facilitated by trained health workers, can provide an accessible and cost-effective strategy for follow-up care, particularly in rural areas with limited access to clinics [32]. Furthermore, disaggregating national health data by demographic groups enables the development of responsive and equitable programming. Targeted interventions and nutrition-sensitive policies should be developed to address the socioeconomic determinants of diet and lifestyle.

Interventions to promote healthy, balanced diets, particularly fruit and vegetable intake, are urgently needed. Mass media campaigns promoting healthy dietary practices and highlighting their association with hypertension and diabetes are recommended. Place-of-residence-specific interventions should be incorporated into national policy formulation to address urban-rural disparities. Prioritizing these specific interventions in national policy formulation will help Bangladesh fulfill its commitment to the 2030 UN Sustainable Development Goals [84]. The national health policies of Bangladesh, particularly those addressing hypertension, should be guided by the World Health Organization’s evidence-based recommendations for the initiation and management of hypertension treatment in adults [84].

The major recommendations in the WHO guidelines include the following: i) developing a standardized treatment protocol through adopting a simplified algorithm-based treatment protocol to ensure consistency and scalability; ii) lowering treatment thresholds by initiating pharmacological treatment at >140/90 mmHg for most adults, and at lower thresholds for those with higher cardiovascular risk; iii) monitoring and establishing regular follow-up intervals and clear targets for blood pressure control (generally >140/90 mmHg); iv) leveraging mobile health and telemedicine to support adherence and remote monitoring; and v) developing equity-focused strategies by addressing social determinants and ensuring access for disadvantaged populations.

### Contributions and limitations of the study

The major strength of this study was its population-based survey design, which enabled a large sample comprising men and women from eight divisions in Bangladesh. As such, the results presented herein are generalizable to Bangladesh’s rural adult population. Furthermore, the findings of this work are detailed and comprehensive, as the collected data consisted of both individual- and household-level components. Moreover, the use of standardized physical examinations to determine the participants’ height, weight, and blood pressure mitigated recall bias and enhanced the accuracy of the findings. In addition to appropriate statistical methods for estimating the age-standardized prevalence of hypertension risk factors in the population, principal component analysis was used to determine the wealth quintiles and, thus, the SES of the surveyed households.

Our study has several limitations. First, our study employed a cross-sectional design, which limits our ability to identify causal relationships between hypertension and the selected risk factors over time, as opposed to longitudinal data. Second, within the scope of our study, only household heads and their spouses were included; other household members, particularly elderly members, were excluded. This limited scope may have introduced selection bias. Future studies should include all adult household members. Third, because our study measured blood pressure in a single arm, this may have introduced measurement bias. Fourth, the data on physical activity were obtained retrospectively, with participants required to recall several activity scenarios in minutes/hours/days/weeks. As a result, participants may have under- or overestimated their physical activity levels, leading to recall bias. Similarly, questions about adequate vegetable and fruit intake may have been difficult for participants to conceptualize, as recalling one’s intake over a typical week/day is highly context-dependent and influenced by temporal food availability and affordability. Furthermore, variations in cultural practices, dietary habits, socioeconomic status, and education levels across districts can differentially influence hypertension risk factors. Thus, future research could develop and incorporate objective measures to enhance the validity of these variables.

## Conclusion

Consistent with prior studies, our findings revealed that one-third of Bangladesh’s rural adult population is living with elevated blood pressure, with hypertension rates being highest among older men and women. High blood pressure was also found to be associated with inadequate intake of fruits and vegetables. Obesity and overweight were also found to significantly influence the prevalence of hypertension in the study sample. While prior studies have shown tobacco consumption and educational attainment to be important risk factors among both sexes and women, respectively, our findings could not establish a significant positive association between smoking and hypertension. However, unadjusted analysis indicated some degree of correlation between tobacco consumption and hypertension. Results also showed no significant association between education and hypertension among women. Moreover, although our findings show no direct association between SES and hypertension prevalence, they did show a clear link between SES and hypertension via various related behavioral and physical risk factors.

Our findings contribute to the existing understanding of hypertension epidemiology in rural Bangladesh by demonstrating the multi-layered and interconnected nature of the risk factors. While access to information and health resources may reduce risk factors among advantaged individuals, the relationship between SES and risk factors underscores the need for equity-based techniques to minimize their impact.

Targeted efforts should focus on promoting healthy aging strategies, enhancing access to cardiovascular screening and management, and advocating for lifestyle modifications, including increased physical activity and healthier dietary practices. Customized interventions that consider sex-specific risk factors and socioeconomic contexts are essential to reducing the prevalence of hypertension in this population effectively. Additionally, addressing rural-urban disparities in healthcare services, infrastructure, and socioeconomic conditions requires serious policy attention and effective interventions.

## Supporting information

S1 File Inclusivity questionnaire. Filled-out inclusivity in global research questionnaire. (DOCX)
